# POD Nanozyme optimized by charge separation engineering for light/pH activated bacteria catalytic/photodynamic therapy

**DOI:** 10.1038/s41392-022-00900-8

**Published:** 2022-03-28

**Authors:** Changyu Cao, Tingbo Zhang, Nan Yang, Xianghong Niu, Zhaobo Zhou, Jinlan Wang, Dongliang Yang, Peng Chen, Liping Zhong, Xiaochen Dong, Yongxiang Zhao

**Affiliations:** 1 Key Laboratory of Flexible Electronics (KLOFE) and Institute of Advanced Materials (IAM), School of Physical and Mathematical Sciences, Nanjing Tech University (NanjingTech), Nanjing, 211816 China; 2 School of Physics, Southeast University, Nanjing, 211189 China; 3 School of Chemical and Biomedical Engineering, Nanyang Technological University, 62 Nanyang Drive, Singapore, 637459 Singapore; 4 National Center for International Biotargeting Theranostics, Guangxi Key Laboratory of Biotargeting Theranostics, Collaborative Innovation Center for Targeting Tumor Theranostics, Guangxi Medical University, Nanning, Guangxi 530021 China

**Keywords:** Medicinal chemistry, Therapeutics, Health care

## Abstract

The current feasibility of nanocatalysts in clinical anti-infection therapy, especially for drug-resistant bacteria infection is extremely restrained because of the insufficient reactive oxygen generation. Herein, a novel Ag/Bi_2_MoO_6_ (Ag/BMO) nanozyme optimized by charge separation engineering with photoactivated sustainable peroxidase-mimicking activities and NIR-II photodynamic performance was synthesized by solvothermal reaction and photoreduction. The Ag/BMO nanozyme held satisfactory bactericidal performance against methicillin-resistant *Staphylococcus aureus* (MRSA) (~99.9%). The excellent antibacterial performance of Ag/BMO NPs was ascribed to the corporation of peroxidase-like activity, NIR-II photodynamic behavior, and acidity-enhanced release of Ag^+^. As revealed by theoretical calculations, the introduction of Ag to BMO made it easier to separate photo-triggered electron-hole pairs for ROS production. And the conduction and valence band potentials of Ag/BMO NPs were favorable for the reduction of O_2_ to ·O_2_^−^. Under 1064 nm laser irradiation, the electron transfer to BMO was beneficial to the reversible change of Mo^5+^/Mo^6+^, further improving the peroxidase-like catalytic activity and NIR-II photodynamic performance based on the Russell mechanism. In vivo, the Ag/BMO NPs exhibited promising therapeutic effects towards MRSA-infected wounds. This study enriches the nanozyme research and proves that nanozymes can be rationally optimized by charge separation engineering strategy.

## Introduction

Bacterial infection afflicts millions of people annually worldwide, causing a severe threat to public health.^1^ And the emergence of antibiotics greatly relieved the pain of infected patients. However, antibiotic indiscriminate use has hastened the evolution of multidrug-resistant bacterial strains, bringing great dilemma to the antibiotic-dependent treatment.^2^ Even worse, the exploitation of novel antibiotics lags behind the growth of multidrug resistance.^3,4^ Consequently, there is an urgent need to discover novel antibiotic-free antibacterial avenues to solve this issue.

Very recently, nanozyme antibacterial therapy has emerged as a potential treatment strategy harboring broad-spectrum antibacterial activity.^5–8^ Typically, nanozyme antibacterial therapy can kill bacteria by employing artificial nanomaterials with inherent enzyme-like activity, for instance, catalyzing the formation of cytotoxic hydroxyl radical (·OH) from hydrogen peroxide (H_2_O_2_).^9–12^ Subsequently, these generated ROS like ·OH can attack and destroy bacterial membrane, DNA, and protein at the acidic infectious site, further resulting in bacterial inactivation.^13,14^ Notably, compared with antibiotics, the bactericidal strategy based on the production of reactive oxygen species (ROS) can significantly avoid the occurrence of drug resistance.^15,16^ Currently, various nanozymes including carbon and metal oxide/chalcogenide nanomaterials, have been successfully employed as antibacterial agents. Nevertheless, the insufficient catalytic activity of nanozymes makes achieving desirable antibacterial efficiency problematic.^17^

Bi_2_MoO_6_ is an inorganic semiconductor photocatalyst and has been widely applied in pollutant removal, biosensor, and so on.^18–22^ However, the catalytic activity of pure Bi_2_MoO_6_ is finite owing to its rapid electron-hole recombination rate and low carrier mobility. To address these deficiencies, plasmonic metal materials (for example, Au, Ag) that commonly possess negative permittivity, have been introduced to improve the catalytic performance with the aid of visible light by the increment of charge separation lifetimes and interfacial charge transfer capability.^23,24^ However, the visible light with poor penetration depth hinders the application of Bi_2_MoO_6_ in the biomedical field. In contrast, NIR-II light with greater tissue penetration and higher maximum allowable exposure exhibit tremendous potential in biomedical photonics.^25^ Therefore, we conceive that the development of NIR-II activated nanozyme based on Bi_2_MoO_6_ composite optimized by charge separation engineering shall be the game-changers.

Herein, Ag/Bi_2_MoO_6_ NPs (Ag/BMO NPs) with enhanced catalytic activity under NIR-II light were prepared for synergistic nanozyme antibacterial therapy and NIR-II photodynamic antibacterial therapy (PDAT). With the introduction of Ag, engineered Ag/BMO NPs exhibit a strong NIR-II absorption. Enzyme-like activity analysis confirmed that Ag/BMO nanozyme exhibited enhanced peroxidase-like catalytic performance under NIR-II light. Besides, a large amount of singlet oxygen is produced for NIR-II PDAT when treated by a 1064 nm laser. All antibacterial results demonstrated that Ag/BMO NPs possessing satisfactory biocompatibility can eradicate methicillin-resistant *Staphylococcus aureus* (MRSA) effectively with the assistance of a 1064 nm laser. Further, the working mechanisms underlying the synergy of self-replenishment, sustainability, and coupling of the cascaded nanocatalytic reactions are carefully revealed via the density functional theory (DFT) calculation. This study demonstrates that Ag/BMO NPs nanozyme with NIR-II enhanced peroxidase-like property and NIR-II light-activated PDT has a great prospect in the fields of anti-infective therapy.

## Results

### Preparation and characterization of Ag/BMO NPs

**Scheme 1:**
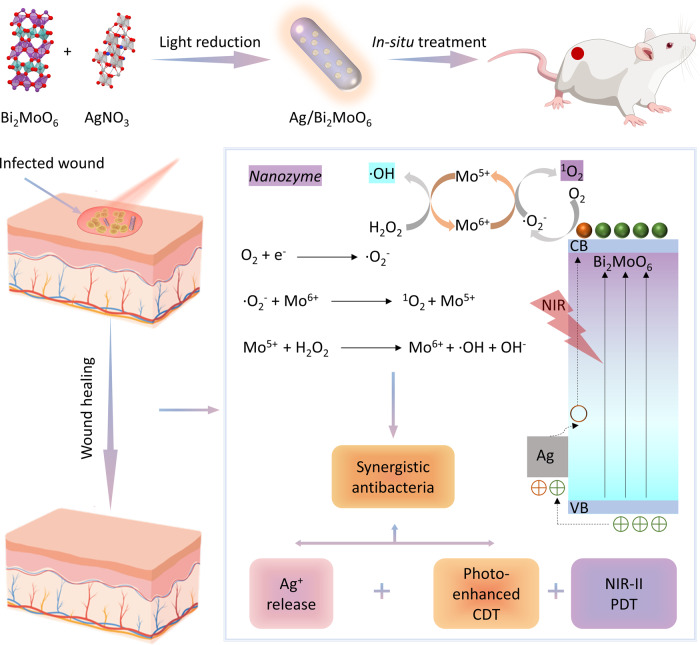
Preparation of Ag/BMO nanozyme and NIR-enhanced catalytic activity mechanisms for synergistic bacterial therapy

**Fig. 1:**
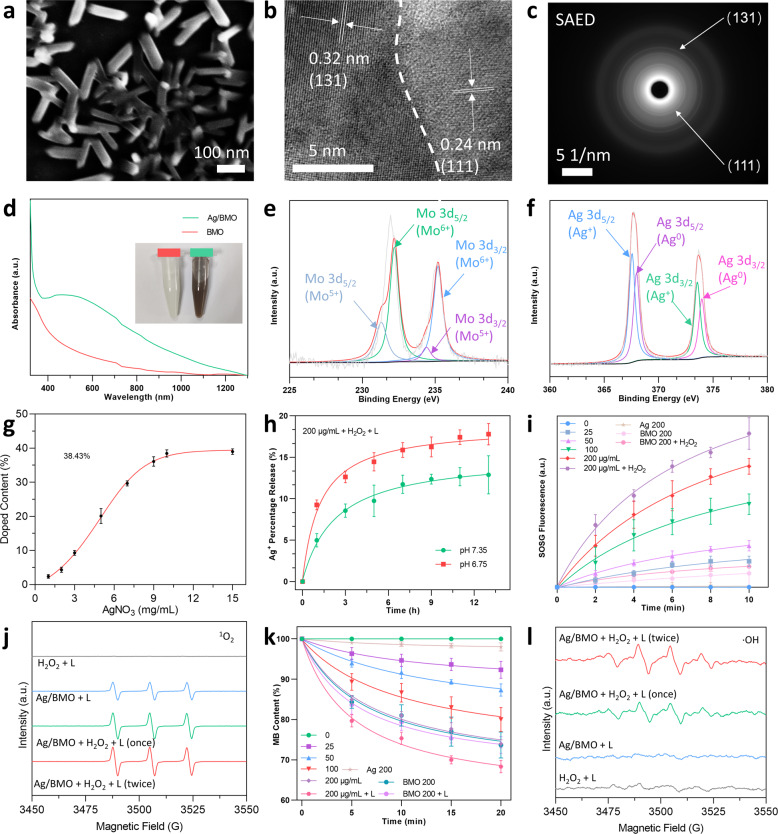
Characterization, Ag+ release, and photo-enhanced ROS generation ability of Ag/BMO. If not otherwise specified, 1064 nm laser (1 W cm^−2^, 10 min) was applied in the laser treatment groups and all NPs were dissolved in DI water for detection. **a**–**c** SEM, HRTEM, SAED images of Ag/BMO NPs. **d** UV-vis absorption of BMO and Ag/BMO NPs solution in water. **e**, **f** XPS spectra of Mo 3d and Ag 3d. **g** Doping content of Ag NPs (The concentration of AgNO_3_ is ranging from 1 to 15 mg L^−1^). **h** Percentage release of Ag^+^ at pH 7.35 and pH 6.75. **i** The ^1^O_2_ detection using SOSG probe. **j** ESR spectra of ^1^O_2_. **k** The ·OH detection using MB indicator. **I** ESR spectra of ·OH

Ag/BMO NPs were fabricated using a two-step synthesis that included solvothermal synthesis and photoreduction as exhibited in Scheme 1. The PVP modified Ag/BMO NPs are highly dispersed in an aqueous solution. As exhibited in Fig. 1a, scanning electron microscopy (SEM) revealed that Ag/BMO NPs possess an average length of ~116 nm with an aspect ratio of ~3 (*n* = 50). Two crystalline lattice spacings could be observed at 0.32 and 0.24 nm shown in the HRTEM image, which corresponds to (131) and (111) planes of BMO and Ag NPs, respectively (Fig. 1b). Moreover, the high-index planes of NPs were also evidenced by the SAED (selected area electron diffraction) pattern shown in Fig. 1c.

The introduction of conductive metals can greatly narrow the effective bandgap, thus extending the absorption spectrum of BMO NPs from ultraviolet to NIR-I and NIR-II regions (Fig. 1d). The NIR-II laser outperforms the NIR-I laser in clinic applications because of its deeper tissue penetration depth and higher MPE (maximum permissible exposure). As a result, a 1064 nm laser was applied in subsequent experiments. Whereafter, the chemical valence was studied using X-ray photoelectron spectroscopy (XPS). Peaks of Mo^5+^ at 231.1 eV and 234.2 eV and peaks of Mo^6+^ at 232.2 eV and 235.2 eV could be deconvolved in Fig. 1e. Peaks of Ag^+^ at 367.4 eV and 373.6 eV and peaks of Ag^0^ at 368.1 eV and 374.0 eV could also be found in Ag’s XPS spectrum (Fig. 1f). The existence of Mo^6+^/Mo^5+^ and Ag^+^/Ag^0^ redox couples may endow the nanoparticle with enzymatic property and antibacterial activity.

### Ag^+^ Release and ROS generation property of Ag/BMO NPs

ICP-MS was used to assess the Ag doping rate,^26–28^, which reached a maximum of ~38.43% when the AgNO_3_ feeding concentration was increased to 10 mg mL^−1^ (Fig. 1g). In Fig. 1h, the release behavior of Ag^+^ at various pH values was also investigated. Under neutral conditions (pH 7.35), 12.88% of Ag^+^ was released for 13 h, while the release amount increased to 17.78% at acidic conditions (pH 6.75, mimicking infected microenvironment). This phenomenon can be ascribed to that the acidity accelerates oxidation of Ag NPs (4Ag + O_2_ + 4H^+^ = 4Ag^+^ + 2H_2_O).^29^ Hence, in an infection acidic microenvironment, the release of Ag^+^ could be enhanced to achieve a stronger antibacterial effect.

Russell mechanism means that ^1^O_2_ formation can be generated from a linear tetraoxide reduction in the presence of metal ions (like Mo^6+^)^30^ and Fenton-like catalytic activity indicates the conversion from H_2_O_2_ to ·OH induced by Fenton agents (including nanozymes with peroxidase (POD)-like activities).^31^ According to the result of the SOSG probe and electron spin resonance (ESR) test, Ag/BMO NPs could produce a large amount of ^1^O_2_ when exposed to a 1064 nm laser (Fig. 1i, j). As a result, Ag/BMO NPs can be used as a photosensitizer in PDAT. This finding also indicated that the higher concentration of Mo^6+^ would boost the formation of ^1^O_2_ via the Russell mechanism, with the help of the Fenton-like reaction and reduction of O_2_.^30,32^

Furthermore, the POD-like activity of Ag/BMO nanozyme was investigated using methylene blue (MB), whose light adsorption diminishes when exposed to ·OH. As exhibited in Fig. 1k, laser stimulation enhances the peroxidase (POD)-like activity significantly. Moreover, ESR using the DMPO probe further confirmed the generation of ·OH via Fenton-like reaction in Fig. 1l. Taken together, benefiting from the enhanced release of Ag^+^ and sustainable production of ^1^O_2_ and ·OH capacity, Ag/BMO nanozyme maybe possess great potential in anti-infective therapy.

### Theoretical calculations

**Fig. 2:**
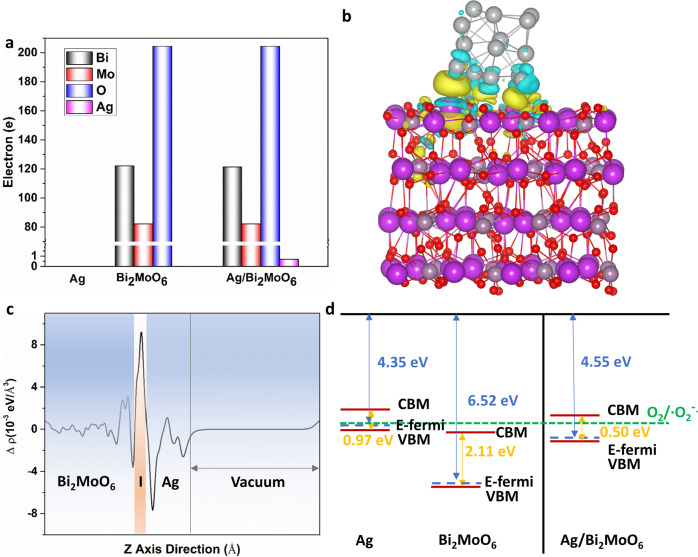
Density functional theory theoretical calculations of Ag/BMO nanozyme. **a** Bader charge of Ag, BMO, and Ag/BMO. **b**, **c** Differential charge density and plane-averaged electron density difference along the *z*-axis of Ag/BMO. The isosurface is 0.002 eV/Å^3^. The yellow and gray color represents gain and lost electrons, respectively. **d** CB and VB configurations of Ag, BMO, and Ag/BMO

Calculation via density functional theory **(**DFT) was further carried out to illustrate the detailed influence of Ag doping on BMO NPs. As presented in Fig. 2 and Supplementary Fig. 1a–c, the bandgap was narrowed from 2.11 eV (BMO NPs) to 0.50 eV (Ag/BMO NPs) by doping Ag, which provided impurity energy level to endow Ag/BMO NPs with robust adsorption in the NIR-II region. In Fig. S1d–g, Ag/BMO NPs had a more localized and concentrated charge distribution at conduction band (CB) and valence band (VB) than BMO NPs. The Ag could transfer 0.7e to BMO NPs in Ag/BMO NPs interface, making Ag with an electron-deficient state and BMO NPs with an electron-rich state (Fig. 2a–c). More importantly, electrons were more likely to escape from the CB of BMO NPs because of the decreased work function of Ag/BMO NPs from 6.57 to 4.55 eV (Supplementary Fig. 1h–j). Because the position of CB (−0.39 V) and VB (0.11 V) of Ag/BMO NPs matches with the O_2_/·O_2_^−^ reduction potential, the escaped electron could significantly catalyze the reduction of O_2_ to ·O_2_^−^ (Fig. 2d). Taken together, the above analysis revealed that Ag doping decreases the bandgap, creates localized charge distribution, and accelerates the escape of electrons, thus progressing the level of ·O_2_^−^ for enhanced PDAT and chemodynamic antibacterial therapy (CDAT).

### In vitro antibacterial performance of Ag/BMO NPs

**Fig. 3:**
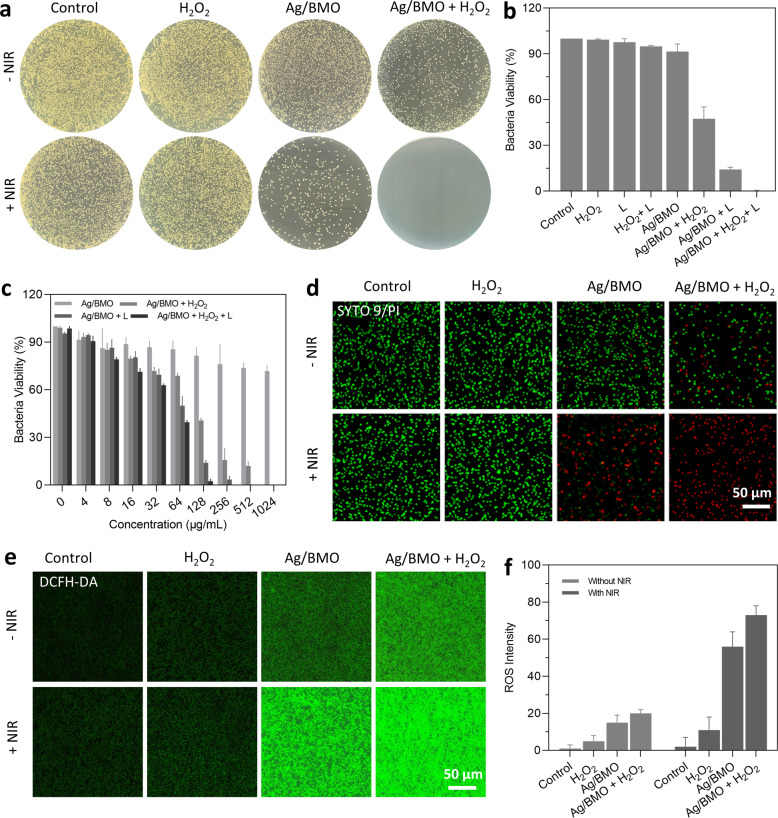
In vitro antibacterial performance of Ag/BMO nanozyme. 1064 nm laser: 1 W cm^−2^ for 10 min, H_2_O_2_: 3 mM, Ag/BMO NPs: 200 μg mL^−1^. If not otherwise specified, all NPs were dissolved in DI water for detection. **a** Plate photographs demonstrating the antibacterial activity of Ag/BMO NPs. **b** Corresponding quantitive survival ratio of MRSA in (**a**). **c** Bacteria viability by monitoring absorbance change at 600 nm (The concentration of NPs solution ranged from 0 to 1024 μg mL^−1^). **d** CLSM images of MRSA stained by LIVE/DEAD dye following incubation with Ag/BMO NPs with or without NIR laser irradiation. (PI emits red fluorescence and SYTO9 emits green fluorescence). **e**, **f** Fluorescence images and the corresponding quantified analysis result using DCFH-DA fluorescent probe

Encouraged by the NIR-II light boosted Fenton-like catalytic property (·OH), the NIR-II light-induced photodynamic property (^1^O_2_), and acidity accelerated Ag^+^ release behavior of the as-prepared Ag/BMO NPs, the standard plate counting assay was performed to evaluate the bactericidal activity of Ag/BMO NPs against MRSA. With the addition of laser irradiation or H_2_O_2_, the bactericidal effect was considerably improved (Fig. 3a, b). All MRSA were killed upon exposure to Ag/BMO NPs (200 μg mL^−1^), 1064 nm laser (1 W cm^−2^, 10 min), and H_2_O_2_ (3 mM). The result was also verified using the broth microdilution method (Fig. 3c).^33^ In the presence or absence of laser irradiation, the bactericidal activity of Ag/BMO NPs was dose-dependent. The combination index (CI) of CDAT and PDAT was calculated to be 0.57 (<0.6), indicating the outstanding synergetic antibacterial efficiency.^34^ Live/dead bacteria staining experiment (Fig. 3d, Supplementary Fig. 2) and bacterial morphological changes (Supplementary Fig. 3) further confirmed the synergetic effects of CDAT and PDAT towards MRSA. A cell-permeable fluorescent probe DCFH-DA was employed to assess the intracellular ROS level. Consistently, with the aid of laser and H_2_O_2_ treatment, the bacteria incubated with Ag/BMO NPs emitted a strong fluorescent signal (Fig. 3e, f) due to the high generation of ROS. These above-mentioned results confirmed that the Ag/BMO NPs with photo-enhanced ROS generation ability hold significant potential for the efficient elimination of multidrug-resistant bacteria.

### In vitro cytotoxicity and hemolysis evaluation

Biomaterials with good biocompatibility are favorable in biomedical applications. Before the antibacterial performance evaluation in vivo, the cytocompatibility and hemocompatibility were implemented. First, the in vitro cytotoxicity of Ag/BMO NPs was evaluated using an MTT assay. Herein, human umbilical vein endothelial cells (HUVECs) and human keratinocytes cells (HaCaTs) were chosen as the study model. As shown in Supplementary Fig. 4, even when the concentration of NP rose to 200 μg mL^−1^, all cell viabilities remained greater than 80%. Then, the erythrocytes of mice were extracted to assess the hemolytic activity of Ag/BMO NPs (Supplementary Fig. 5). In PBS and different concentrations of Ag/BMO NPs solution (25–200 μg mL^−1^), the supernatants were transparent and colorless, and the relative hemolysis ratios of Ag/BMO NPs were significantly lower than the allowable limit (5%). On the contrary, the supernatant treated with water was bright red. This result indicated that Ag/BMO NPs possessed satisfactory biocompatibility, which could be used for the therapy of wound infection in vivo.

### In vivo treatment of wound infection

**Fig. 4:**
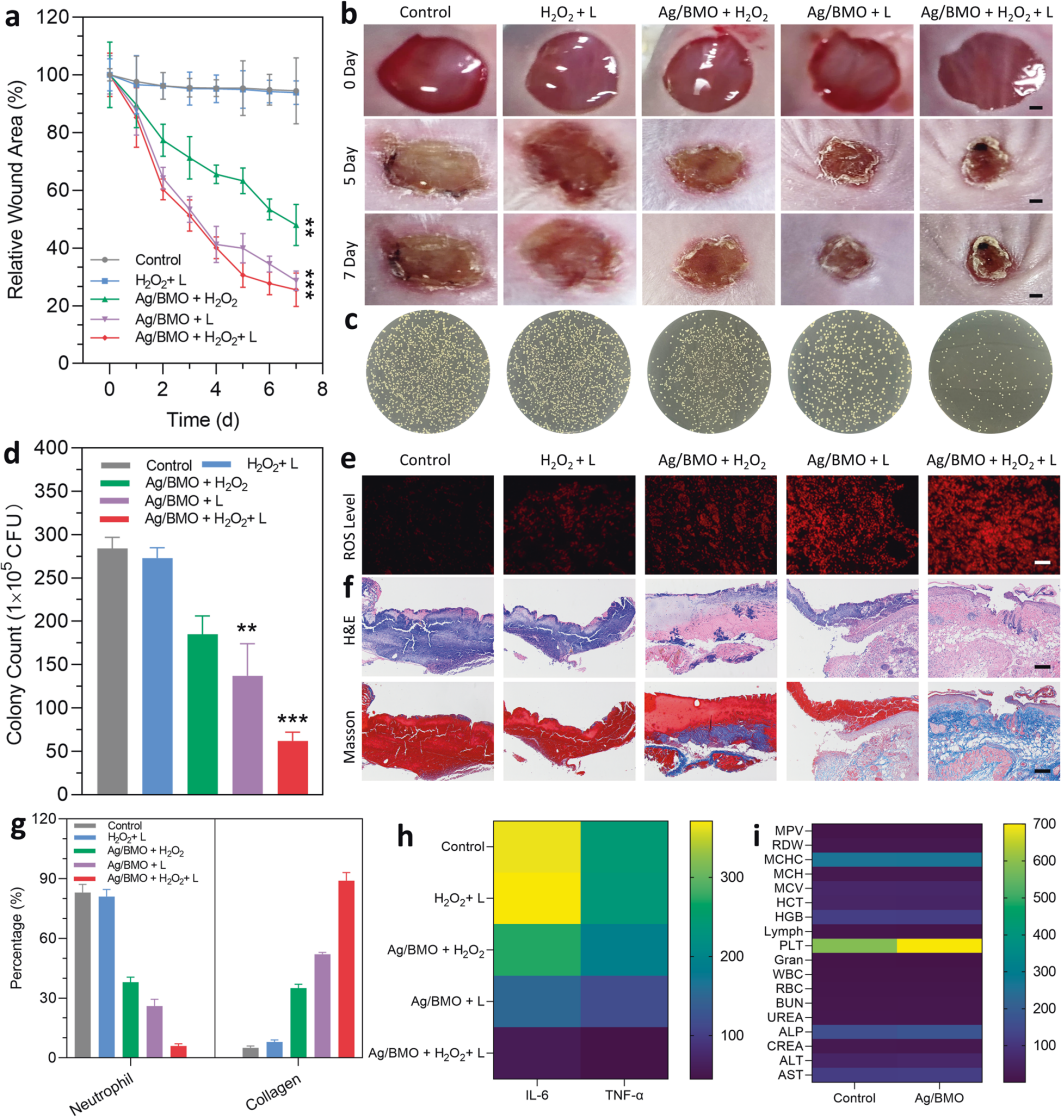
In vivo MRSA-infected wound healing effect of Ag/BMO nanozyme. 1064 nm laser: 1 W cm^−2^ for 10 min, H_2_O_2_: 3 mM, Ag/BMO NPs: 200 μg mL^−1^. If not otherwise specified, all NPs were dissolved in DI water for detection. **a** The change of wound areas for 7 d. *P*-value indicates the significant difference. **P < 0.01, ***P < 0.001. **b**, **c** Photographs of MRSA-infected wounds in various groups and the corresponding plates after treatments. Scar bar: 1 mm. **d** The quantified data of survival MRSA in infected wounds treated with different groups. *P*-value indicates the significant difference. **P < 0.01, ***P < 0.001. **e** ROS level of infected wounds (more red fluorescence indicated more ROS content). **f** H&E and Masson-stained tissues slices of infected wounds. **g** The percentage number of neutrophils and collagen index. **h** Levels of IL-6 and TNF-α. **i** Blood biochemistry and physiological index analysis for control and Ag/BMO groups

In vivo therapeutic effectiveness was further studied using the MRSA-infected Balb/c mice model. In Fig. 4a, b, and Supplementary Fig. 6, without laser treatment, the non-sustainable CDAT of Ag/BMO NPs could accelerate wound healing to a limited extent. In comparison, the wound receiving laser irradiation significantly improved wound healing (70% reduction compared to control). Besides, with the addition of H_2_O_2_, the best elimination of MRSA in 7 days was then achieved (Fig. 4c, d), demonstrating the exceptional potency of Ag/BMO NPs because of the sustained and synergistic coupling of CDAT and PDAT.

To determine the ROS level, wound tissues were harvested and stained with dihydroethidium (DHE). As expected, under laser irradiation, the ROS level in the Ag/BMO NPs treatment wound increased due to the occurrence of photo activated photodynamic behavior, while the ROS level in the wound treated with Ag/BMO NPs + H_2_O_2_ was the highest due to the ·OH generation of CDAT and the ^1^O_2_ production of PDAT (Fig. 4e).

Further, infected wounds were also extracted and sectioned for histomorphological investigation. As exhibited in Fig. 4f, g, a large number of neutrophils and few collagen depositions were found in the PBS and H_2_O_2_ + L groups. With the introduction of H_2_O_2_ or laser, the treatment effects were improved to a certain degree, especially for Ag/BMO + L group, the percentage of neutrophils decreased to 26% and the collagen index increased to 52%. More importantly, the least neutrophils (6%) and highest collagen index (89%) were observed in the synergistic group (Ag/BMO + H_2_O_2_ + L group), demonstrating a favorable reconstitution of extracellular matrix and desirable tissue regeneration.

More importantly, the inflammatory response was carefully monitored using an enzyme-linked immune sorbent test (ELISA) to track changes of typical inflammatory cytokines including TNF-α (tumor necrosis factor-α) and IL-6 (interleukin-6). As displayed in Fig. 4h, a significant drop of TNF-α and IL-6 were found in comparison with the PBS group at day 7 post-therapy, showing that the inflammatory response in the Ag/BMO + H_2_O_2_ + L group was significantly reduced. This could be mainly contributed to the germicidal effect of ROS and Ag^+^ produced from Ag/BMO NPs. Moreover, the systemic inflammatory response was assessed through the blood analysis. Compared with the PBS group, the white blood cell (WBC) and granulocyte were decreased to almost normal levels (Fig. 4i). These results demonstrated that Ag/BMO NPs played outstanding “antibiotics” for efficient bacteria-killing and wound healing with the assistant of H_2_O_2_ and laser.

Even after receiving laser treatment, the mouse body weight did not show significant affection after administration with Ag/BMO NPs (Supplementary Fig. 7). According to the H&E staining assay, no apparent abnormality was found in the main organs (Supplementary Fig. 8). Following the treatments, the blood biochemistry assay and blood routine examination also revealed no abnormalities (Fig. 4i). Taken together, during the therapy, Ag/BMO NPs didn’t exhibit apparent toxicity.

## Discussion

The emergence of multidrug resistance is one of the most concerns for anti-infection therapy.^35^ From this angle, nanozyme catalytic therapy based on reactive oxygen species shall be game-changer.^5–8^ In this work, Ag/BMO nanozyme was prepared through solvothermal and photoreduction routes. The absorbance spectrum of Ag/BMO showed that the NPs synthesized by this method exhibited an enhanced absorption in the NIR-II region, enabling them to be excited by the NIR-II laser irradiation for PDAT. In addition, XPS data showed that Mo^6+^/Mo^5+^ and Ag^+^/Ag^0^ redox couples were simultaneously presented in NPs, indicating that Ag/BMO nanozymes not only possess enzymatic property but also inherent antibacterial activity.^36^

The release profiles shown in Fig. 1h demonstrated that an enhanced release behavior of Ag^+^ would occur in an acid environment,^29^ which is of great benefit to achieve a more specific anti-infection therapy. In addition, a boosting ROS generation capacity of Ag/BMO nanozyme was observed when introducing H_2_O_2_ and laser, confirming that NP could effectively produce ROS for bacteria-killing owing to the photodynamic effect and POD enzyme-mimicking property.^30–32^ Theoretical calculations via DFT carried out in Fig. 2 revealed the ROS generation mechanism of Ag/BMO nanozyme. These data revealed that Ag doping in BMO NP decreased the bandgap, created localized charge distribution, and accelerated the escape of electrons, thus progressing the level of ·O_2_^−^ for enhanced PDAT and CDAT. Furthermore, as shown in Fig. 3a–d, the bacterial plate counting assay and live/dead staining assay showed that the best antibacterial performance occurred in the synergistic group. And over 99% of bacteria were killed in the Ag/BMO + H_2_O_2_ + L group, affording great potential for in vivo disinfection. Additionally, DCFH staining further confirmed that the achievement of desirable antibacterial performance was attributed to the generation of boost ROS (Fig. 3e, f).

Encouraged by the results of in vitro assay, in vivo assay was also performed using the MRSA-infected Balb/c mice model shown in Fig. 4. The best wound repairing performance was observed in the synergistic treatment group with the least wound area, bacterial residue, and inflammatory response. Furthermore, the mouse body weight did not show significant affection after administration with Ag/BMO NPs and no apparent abnormality was found in the main organs. The blood biochemistry assay and blood routine examination also revealed no abnormalities. Taken together, during the therapy, Ag/BMO NPs didn’t exhibit apparent toxicity.

In conclusion, a nanozyme (Ag/BMO NPs) with NIR-II light enhanced peroxidase-like activity and NIR-II PDAT was prepared. Optimized by charge separation engineering, the Ag/BMO NPs exhibit a narrowed bandgap with boosted photocatalytic capability in the NIR-II region, and replenishable catalytic capacity because of the presence of Mo^6+^/Mo^5+^ redox sites. Ag/BMO NPs exhibit outstanding chemodynamic and photodynamic synergistic capabilities with negligible biotoxicity according to these in vitro and in vivo assays. The outstanding antibacterial activity is benefited from the chemodynamic and photodynamic synergistic antibacterial therapy, as well as the enhanced ROS production performance via sustainably coupled catalytic reactions. We believe that nanozymes with excellent photo-enhanced enzyme-like activity and sustainable generation of ^1^O_2_ and ·OH capacity can be used as alternative antibiotics for treating infectious disorders.

## Materials and methods

### Materials

Bi(NO_3_)_3_·5H_2_O, Na_2_MoO_4_·2H_2_O, methylene blue (MB), and AgNO_3_ were ordered from Sigma-Aldrich. 2,7-dichlorofluorescin diacetate (DCFH-DA) was purchased from Aladdin. Poly(vinylpyrrolidone) (M_w_ = 58,000), propanediol, glycol, sodium acetate, absolute ethanol (EtOH), dimethyl sulfoxide (DMSO), and sodium hydroxide (NaOH) were offered by Adamas-beta. Singlet Oxygen Sensor Green (SOSG) and Live/Dead Bacterial Viability Kits were ordered from Thermo Fisher. MTT, HUVECs, HaCaTs, DMEM (Dulbecco’s modified eagle’s medium), and PBS (phosphate-buffered saline) were purchased from Beyotime Biotech. MRSA (ATCC43300) was available from Promega. Before usage, LB liquid medium was autoclaved for 20 min at 120 °C. Milli-Q deionized (DI) water was used during the whole experiment. All used solvents are DI water if not specifically mentioned.

### Characterization

To describe the structure and morphology of the NPs, TEM (HT7700, Hitachi, Japan), HRTEM, and SEM (S-4800, Hitachi, Japan) were used. XPS (Thermal Scientific K-Alpha instrument, USA) revealed the electronic states of Mo and Ag. CLSM (confocal laser scanning microscope, Olympus, Japan) was used to collect fluorescence images. Ultraviolet-visible absorption spectrometry (Shimadzu, Japan) was employed to collect the absorption spectrum of samples. ICP-MS (inductively coupled plasma spectrometry, Agilent 720ES) was used to determine the content of Ag in NPs.

### Preparation of BMO and Ag/BMO NPs

Under ultrasonic conditions, Na_2_MoO_4_·2H_2_O and Bi(NO_3_)_3_·5H_2_O (0.5 g/1.5 g, m/m) were dissolved in glycol (5 mL), respectively. The above two solutions were then mixed together, and ethanol (35 mL) was added. After stirring (0.5 h), the mixture was treated by the solvothermal method for 10 h at 180 °C. The produced solution was dried at 80 °C for 12 h after being rinsed three times with DI water and ethanol, respectively. The dry powder was then calcined at 450 °C for 2 h, yielding the final BMO NPs. The photoreduction approach was used to prepare Ag/BMO NPs. Under vigorous ultrasound, BMO NP (1 g) was dispersed in ethanol (35 mL). The BMO NPs solution was then treated with 5 mL of AgNO_3_ solution (15 mg mL^−1^ in water). The above-mentioned combination solution was then exposed to a Xe lamp (PL-XQ500 W) for 1 h before being washed three times with ethanol to eliminate the excess AgNO_3_. The solution was then dried (80 °C, 6 h) to yield Ag/BMO NPs for future usage.

### OH detection

To detect the ·OH, different concentrations of Ag/BMO NPs solution (from 0 to 200 μg mL^−1^ in water) were added in MB solution (3 mL, 8 μg mL^−1^ in water). Following that, the mixed solution was stirred for 30 min before H_2_O_2_ (100 μL, 3 mM in water) was then added to it. The mixed solution was centrifuged to extract the NPs after being stirred for 0, 5, 10, 15, and 20 min. The ability to generate ·OH was assessed by the change in absorbance at 660 nm.

### ^1^O_2_ production detection

SOSG (10 μM in DMSO) was employed as a probe to validate the ^1^O_2_ production performance. Varying concentrations of Ag/BMO NPs solution (from 0 to 200 μg mL^−1^ in water) containing SOSG probe (100 μL) were exposed to NIR laser irradiation (1064 nm, 1 W cm^−2^, 10 min). Following that, the ability to generate ^1^O_2_ was measured using a fluorescence spectrophotometer (Ex/Em: 504/525 nm).

### EPR measurement

EPR (Electron paramagnetic resonance) spectroscopy was also performed to test the generation of ROS. In detail, 2,2,6,6-Tetramethyl-1-piperinedinyloxy (TEMPO, 20 μL, 100 mM) was used as the probe to detect ^1^O_2_ and 5,5-dimethyl-1-pyrroline N-oxide (DMPO, 50 μL, 100 mM) was employed as the probe to detect ·OH, respectively. Samples were divided into four groups including H_2_O_2_ + L (control), Ag/BMO + L, Ag/BMO + H_2_O_2_ + L (irradiation for one time) and Ag/BMO + H_2_O_2_ + L (irradiation for two times). Herein, the content of Ag/BMO NPs was 200 μg mL^−1^, the concentration of H_2_O_2_ was 3 mM and the power density of NIR laser was 1 W cm^−2^ (1064 nm, 10 min). After adding DMPO or TEMPO probe to the above-mentioned samples solution, the mixed solution was then transferred into a quartz capillary for EPR measurement at room temperature.

### In vitro cell cytotoxicity assay

MTT assay was performed to assess the biocompatibility of Ag/BMO NPs to HaCaTs or HUVECs. In brief, HaCaTs or HUVECs (100 μL, 10^6^ cells mL^−1^) were first cultured into 96-well plates for 24 h of incubation with varying concentrations of NPs (from 0 to 200 μg mL^−1^ in water) at 37 °C, 5% CO_2_. Following that, for another 4 h of incubation, MTT solution (50 μL, 5 mg mL^−1^) was added. Following a PBS rinse, each well was treated by adding 100 μL of DMSO to disintegrate the formazan precipitation. Finally, the absorbance change at 490 nm of each well was monitored using a microtiter plate reader.

### In vitro hemostatic activity assay

A spectrophotometer was used to assess the hemocompatibility of Ag/BMO NPs. In detail, to obtain the erythrocytes, the whole blood was centrifuged for 10 min (3000 rpm). Following that, the erythrocytes were rinsed three times with PBS and diluted to the final concentration (5% in PBS, v/v). The NPs in various concentrations (500 μL, ranging from 25 to 200 μg mL^−1^) and erythrocyte suspension (500 μL) were then combined in a tube (2 mL) and incubated for 6 h at 37 °C. After centrifugation (10 min, 3000 rpm), the as-obtained supernatant (100 μL) was then put into 96-well culture plates, and the hemoglobin content was quantified by monitoring the absorbance changes at 540 nm using a microplate spectrophotometer. The positive and negative control groups were water and PBS, respectively.

### MRSA culture

MRSA was pre-cultured for 12 h in LB medium at 37 °C. Following that, MRSA was then centrifuged down and gently rinsed three times using PBS solution. MRSA density was measured using an ultraviolet spectrophotometer, and the MRSA was re-dispersed in PBS solution before use.

### In vitro antimicrobial activity of Ag/BMO NPs

Without special mention, the density of the NIR laser was 1 W cm^−2^ (1064 nm, 10 min) and the content of Ag/BMO NPs solution was 200 μg mL^−1^ (in water). The standard plate counting assay was used to assess the antibacterial performance of Ag/BMO NPs. First, bacterial suspension (10^6^ CFU mL^−1^ in PBS, 100 μL) was incubated with Ag/BMO NPs solution (1 mL) for 4 h in the presence or absence of laser irradiation. Following that, MRSA suspension was gently washed with PBS solution before being moved to 96-well plates for an additional 12 h of co-incubation. The absorbances at 600 nm of wells were then measured for analysis. Furthermore, the resultant MRSA suspension (50 μL) was diluted with PBS solution before being evenly distributed on LB plates. The plates were imaged after 24 h incubation at 37 °C, and the colonies were subsequently quantified using ImageJ software. Moreover, the MRSA suspension (10^6^ CFU mL^−1^, 100 μL) was then treated using LIVE/DEAD dyes (3 μL, SYTO9/PI, 1:1, v/v, 15 min) Under light-proof conditions after different treatments with NPs with or without NIR laser irradiation. Following that, all samples were examined using CLSM.

### Intracellular ROS production detection

ROS generation caused by nanozymes was studied using DCFH-DA dye with non-fluorescent and could be converted to green fluorescence DCF by the oxidation of intracellular ROS. In brief, MRSA was seeded in 12-well plates and subsequently cultured for 24 h. Following that, MRSA was treated with PBS, H_2_O_2_, NIR, H_2_O_2_ + L, Ag/BMO, Ag/BMO + H_2_O_2_, Ag/BMO + L, and Ag/BMO + H_2_O_2_ + L, respectively. Inhere, the power density of laser was 1 W cm^−2^ (1064 nm, 10 min), the content of NPs and H_2_O_2_ was 200 μg mL^−1^ and 3 mM, respectively. After 24 h of different treatments, the ROS probe was added to each well for another 30 min incubation. After eliminating any unbound dye, the fluorescence was monitored using the fluorescence microscope.

### Infection model

The animal tests were performed with the permission of Nanjing Tech University, in accordance with applicable laws and guidances. Balb/c mice aged 5–6 weeks old were purchased from Yangzhou University. The dorsal surface of mice was initially depilated in order to create full-thickness wound infection models. Following narcotization, an 8 mm diameter full-thickness incision was established and then MRSA in PBS (10^6^ CFU mL^−1^, 200 μL) was used to infect wounds.

### In vivo wound healing assay

Without special mention, the density of the laser was 1 W cm^−2^ (1064 nm, 10 min), and the content of Ag/BMO and H_2_O_2_ was 200 μg mL^−1^ 3 mM, respectively. Besides, the treatment was only administered once. Mice infected by MRSA were randomly assigned to five groups after one-day infection: PBS, H_2_O_2_ + L, Ag/BMO + H_2_O_2_, Ag/BMO + L, and Ag/BMO + H_2_O_2_ + L. The wounds in the L treatment group were applied with laser irradiation. After 1 or 7 d of various treatments, these infected wounds were then collected and homogenized for bacterial burden analysis. Moreover, these infected wounds were also cemented with paraffin for the pathological slice to assess the inflammation reaction and the healing process of wounds. For H&E staining, the slides were stained by hematoxylin and eosin. For Masson’s trichrome staining, hematoxylin, biebrich scarlet, and aniline blue were used in the dyeing process. The ROS level in wounds after various treatments was determined by dihydroethidium (DHE) staining. The major organs were also taken for pathological investigation. Blood was obtained for routine evaluation in order to measure the extent of inflammatory response.

### Calculation method

The electronic structures of Ag, BMO, and Ag/BMO systems were obtained using density functional theory calculations, which were taken in the VASP.^37^ For the exchange-correlation function, the GGA (generalized gradient approximation) in the form of the PBE (Perdew–Burke–Ernzerhof) approach was used.^38^ The plane wave function’s cutoff energy was set to 400 eV. An Ag/BMO interface (Fig. S1a) comprised of (131) surface of BMO (288 atoms) and Ag cluster (14 atoms) was constructed based on experimental results. 15 Å vacuum layer on the *z*-axis was applied to avoid artificial interaction between adjacent surfaces.

## Supplementary information

supporting information

## Data Availability

All data supported the paper are presented in the paper and/or the Supplementary Materials. The original datasets are also available from the corresponding author on reasonable request.
