# Correction To: POD Nanozyme optimized by charge separation engineering for light/pH activated bacteria catalytic/photodynamic therapy

**DOI:** 10.1038/s41392-023-01476-7

**Published:** 2023-05-24

**Authors:** Changyu Cao, Tingbo Zhang, Nan Yang, Xianghong Niu, Zhaobo Zhou, Jinlan Wang, Dongliang Yang, Peng Chen, Liping Zhong, Xiaochen Dong, Yongxiang Zhao

**Affiliations:** 1 Key Laboratory of Flexible Electronics (KLOFE) and Institute of Advanced Materials (IAM), School of Physical and Mathematical Sciences, Nanjing Tech University (NanjingTech), Nanjing, 211816 China; 2 School of Physics, Southeast University, Nanjing, 211189 China; 3 School of Chemical and Biomedical Engineering, Nanyang Technological University, 62 Nanyang Drive, Singapore, 637459 Singapore; 4 National Center for International Biotargeting Theranostics, Guangxi Key Laboratory of Biotargeting Theranostics, Collaborative Innovation Center for Targeting Tumor Theranostics, Guangxi Medical University, Nanning, Guangxi 530021 China

**Keywords:** Medicinal chemistry, Therapeutics, Health care

Correction to: *Signal Transduction and Targeted Therapy* 10.1038/s41392-022-00900-8, published online 28 March 2022

The plate photo from Control in Figure 4c of the published work was mistakenly used when editing the photos, and the corrected version is demonstrated below. The results and conclusions of this paper were not affected by this error.

**Fig. 4:**
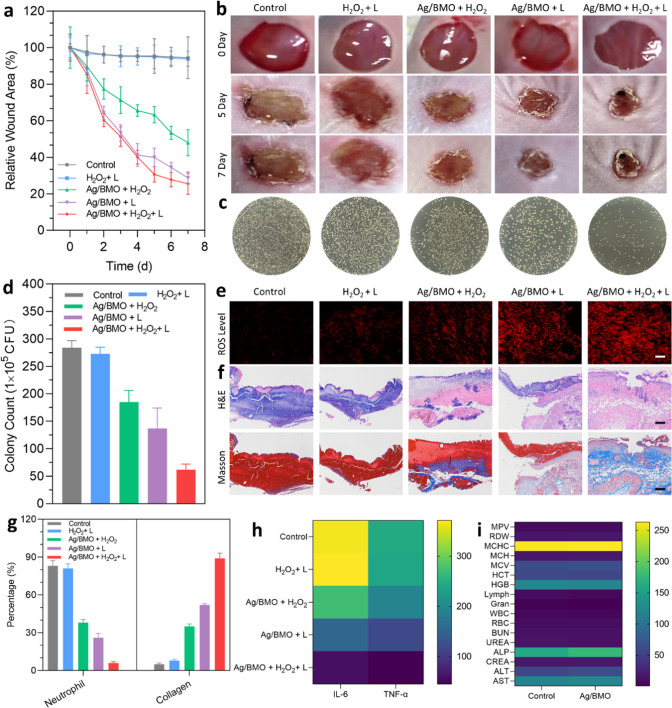
In vivo MRSA-infected wound healing effect of Ag/BMO nanozyme. 1064 nm laser: 1 Wcm^−2^ for 10 min, H_2_O_2_: 3 mM, Ag/BMO NPs: 200 μg mL^−1^. If not otherwise specified, all NPs were dissolved in DI water for detection. **a** The change of wound areas for 7 d. *P*-value indicates the significant difference. **P < 0.01, ***P < 0.001. **b**, **c** Photographs of MRSA-infected wounds in various groups and the corresponding plates after treatments. Scar bar: 1 mm. **d** The quantified data of survival MRSA in infected wounds treated with different groups. *P*-value indicates the significant difference. **P < 0.01, ***P < 0.001. **e** ROS level of infected wounds (more red fluorescence indicated more ROS content). **f** H&E and Masson-stained tissues slices of infected wounds. **g** The percentage number of neutrophils and collagen index. **h** Levels of IL-6 and TNF-α. **i** Blood biochemistry and physiological index analysis for control and Ag/BMO groups

