# Supplementary material for: POD Nanozyme optimized by charge separation engineering for light/pH activated bacteria catalytic/photodynamic therapy
Source: Signal Transduct Target Ther. 2022 Mar 28;7:86. doi: 10.1038/s41392-022-00900-8 (PMC8958166; doi:10.1038/s41392-022-00900-8)
Supplement: Supplementary file 1 — supporting information [file 41392_2022_900_MOESM1_ESM.docx]

Supplementary Materials for

**POD Nanozyme Optimized by Charge Separation Engineering for Light/pH Activated Bacteria Catalytic/Photodynamic Therapy**

Changyu Cao^1#^, Tingbo Zhang^2#^, Nan Yang^1^, Xianghong Niu^2^, Zhaobo Zhou^2^, Jinlan Wang^2^, Dongliang Yang^1^*, Peng Chen^3^, Liping Zhong^4^, Xiaochen Dong^1^*, Yongxiang Zhao^4^*

Correspondence to: Xiaochen Dong ([iamxcdong@njtech.edu.cn](mailto:iamxcdong@njtech.edu.cn)); Dongliang Yang ([yangdl1023@njtech.edu.cn](mailto:yangdl1023@njtech.edu.cn)); Yongxiang Zhao ([yongxiang_zhao@126.com](mailto:yongxiang_zhao@126.com))

**This PDF file includes:**

Figures. S1 to S8

Figure. S1.


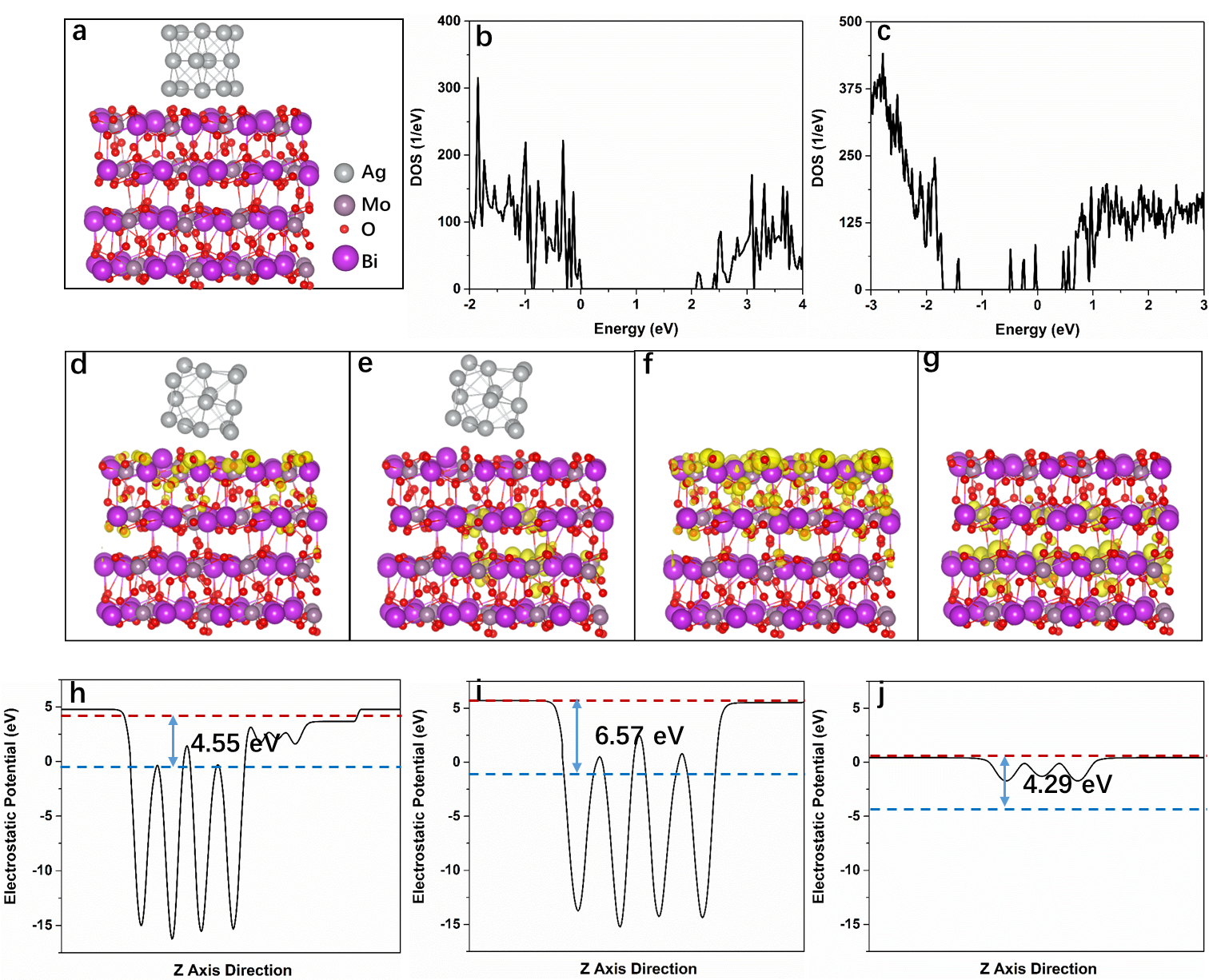


**Figure S1.** (a) Atomic structure of Ag/BMO NPs interface. The DOS of BMO NPs (b) and Ag/BMO NPs (c). The CB and VB of BMO NPs with (d and e) and without (f and g) Ag doping. The isosurface is 0.003 eV/Å^3^. The work function of Ag/BMO NPs (h), BMO NPs (i) and Ag (j).

Figure. S2.


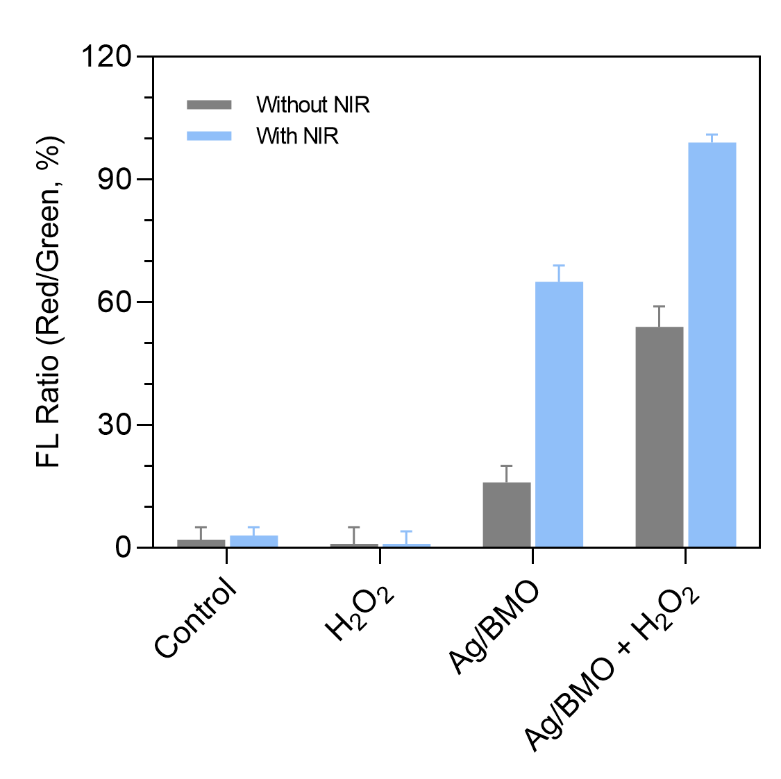


**Figure S2.** The corresponding quantitative analysis of the live/dead fluorescence images in Figure 2d.

Figure. S3.


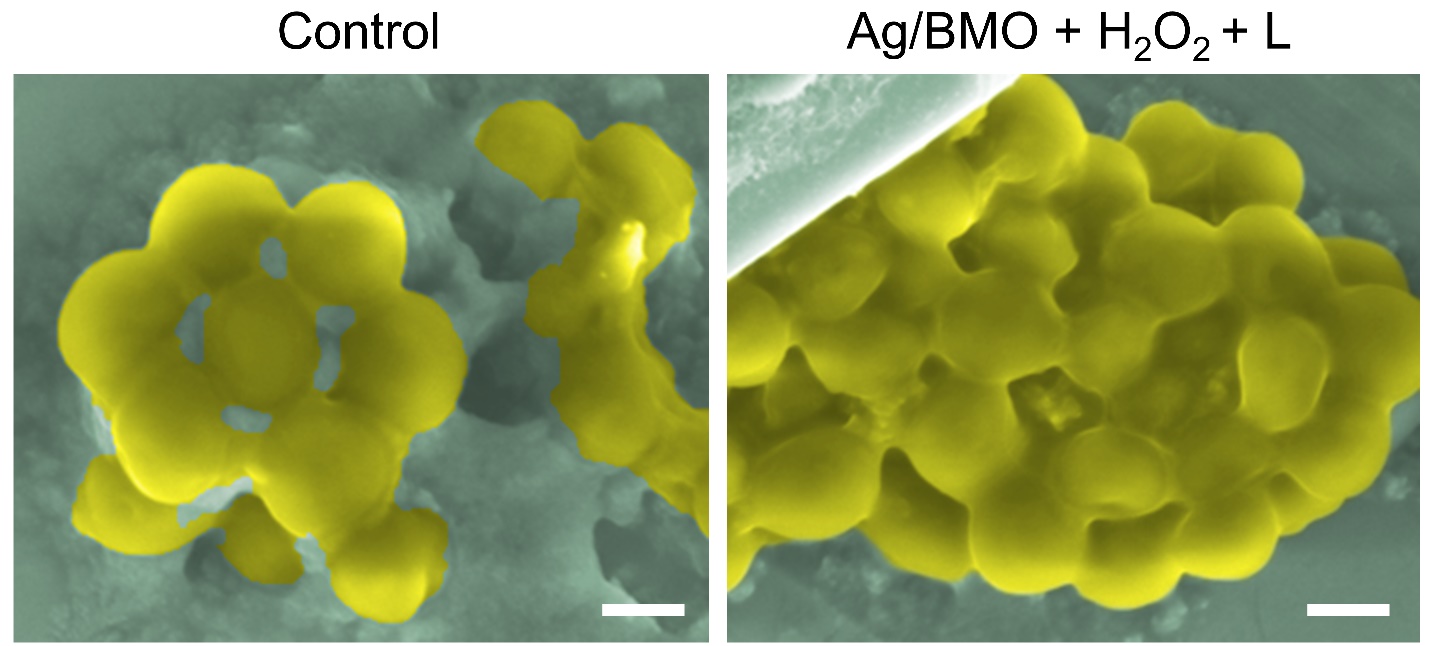


**Figure S3.** All NPs were dissolved in DI water for detection, the content of H_2_O_2_ was 3mM, and the concentration of NPs was 200 μg mL^-1^. SEM images of MRSA treated with Ag/BMO + H_2_O_2_ + L and control groups (1064 nm, 1 W cm^-2^, 10 min). Scar bar: 500 nm.

Figure. S4.

*
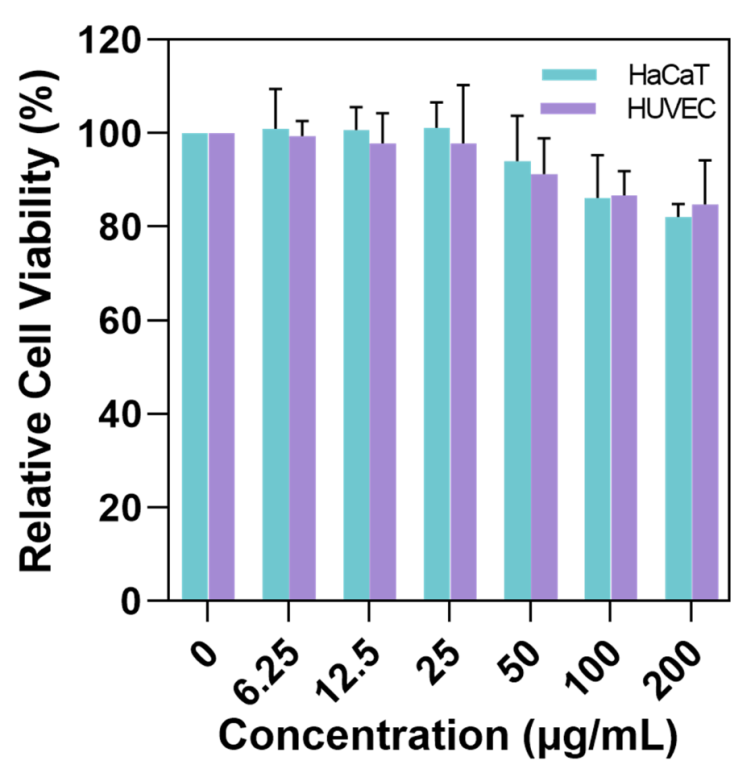
*

**Figure S4.** Relative cell viabilities of HaCaTs and HUVECs treated with different concentrations of Ag/BMO NPs (ranging from 0 to 200 μg mL^-1^).

Figure. S5.

*
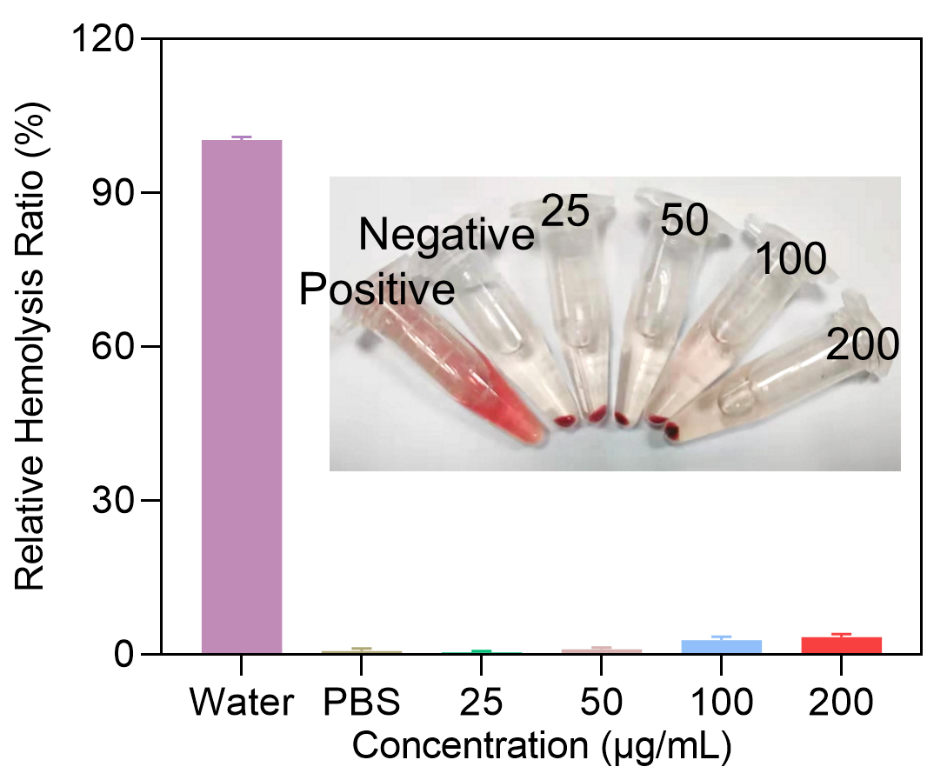
*

**Figure S5.** Relative hemolysis ratio of different concentrations of Ag/BMO NPs (ranging from 25 to 200 μg mL^-1^).

Figure. S6.

*
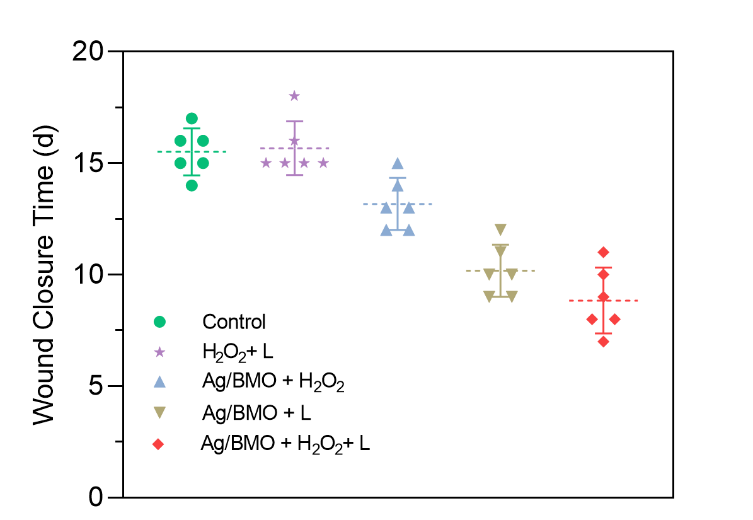
*

**Figure S6.** Wound closure time after different treatments.

Figure. S7.


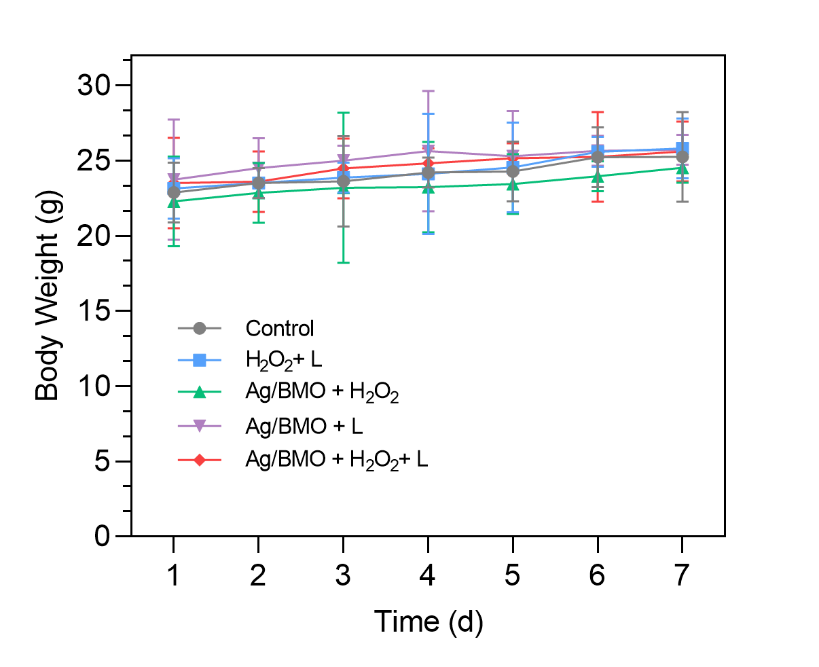


**Figure S7.** Body weight changes of mice after different treatments.

Figure. S8.

*
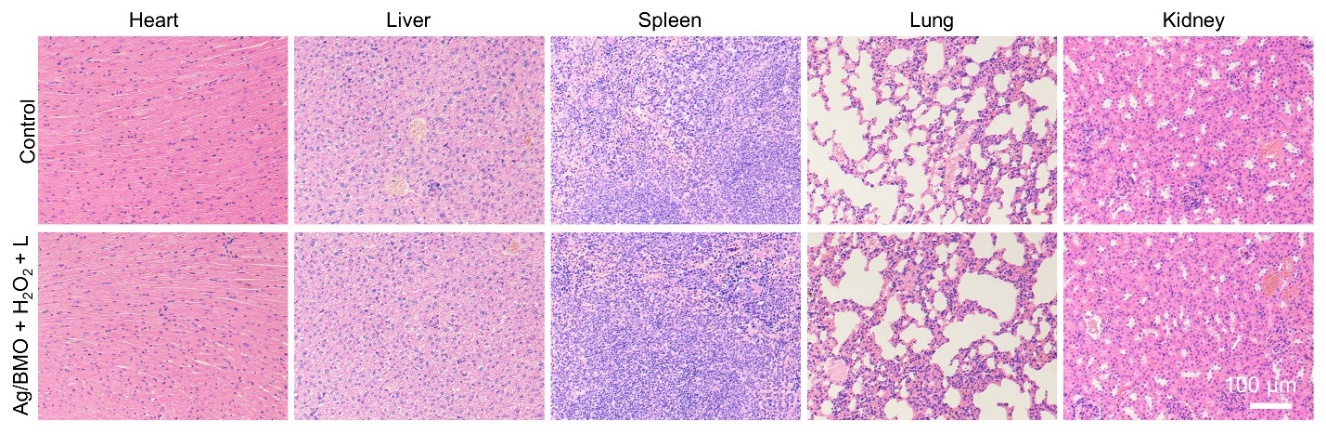
*

**Figure S8.** H&E staining photographs of main organs from the mice with different treatments (7 days).
